# PD1, CTLA4 and TIGIT Expression on T and NK Cells in Granulomatous Diseases: Sarcoidosis and ANCA-Associated Vasculitis

**DOI:** 10.3390/ijms24010256

**Published:** 2022-12-23

**Authors:** Miriana d’Alessandro, Edoardo Conticini, Laura Bergantini, Fabrizio Mezzasalma, Paolo Cameli, Stefano Baglioni, Martina Armati, Marta Abbritti, Elena Bargagli

**Affiliations:** 1 Respiratory Diseases and Lung Transplantation Unit, Department of Medical and Surgical Sciences & Neurosciences, Siena University Hospital, 53100 Siena, Italy; 2 Rheumatology Unit, Department of Medicine, Surgery & Neurosciences, University of Siena, 53100 Siena, Italy; 3 Diagnostic and Interventional Bronchoscopy Unit, Cardio-Thoracic and Vascular Department, University Hospital of Siena Azienda Ospedaliera Universitaria Senese, AOUS, 53100 Siena, Italy; 4 Pneumology Department, Perugia Hospital, 06129 Perugia, Italy

**Keywords:** sarcoidosis, ANCA-associated vasculitis, immune checkpoint molecules

## Abstract

Sarcoidosis is a granulomatous diseases affecting the lungs. Anti-neutrophil cytoplasmic antibody (ANCA)-associated vasculitis (AAV) is a histologically granulomatous B-mediated disorder characterized by activated T cells. The expression of immune checkpoint (IC) molecules (PD1, CTLA4, TIGIT) on T- and NK-cells negatively regulate the T-cell immune function. The present study aimed to explore the peripheral distribution of IC molecules to better elucidate their peripheral tolerance failure, which might reflect the development of diseases. Patients referred to Respiratory Diseases and Rheumatology Unit of Siena University Hospital were prospectively and consecutively enrolled. Healthy subjects were also enrolled as a control group. Multicolor flow cytometric analysis was performed to detect IC molecules in the peripheral blood of patients. Twenty-three patients were consecutively and prospectively enrolled in the study: 11 patients had an AAV diagnosis and 12 had sarcoidosis. CD4^+^PD1^+^ cells were higher in sarcoidosis and GPA than in HC (*p* = 0.0250 and *p* = 0.0253, respectively). CD56^+^CTLA4^+^ were higher in sarcoidosis than GPA, MPA and HC (*p* = 0.0085, *p* = 0.0042 and *p* = 0.0004, respectively). CTLA4^+^NK cells clustered for 100% of sarcoidosis patients according to decision tree analysis, while PD1^+^CD4 and CD8 cells for clustered for 100% of GPA patients. Our analyses showed substantial differences between sarcoidosis and AAV, further confirming the immunological peculiarity of this disease. Despite these advances, the pathogenesis remains incompletely understood, indicating an urgent need for further research to reveal the distinct immunological events in this process, with the hope to open up new therapeutic avenues and, if possible, to develop preventive measures.

## 1. Introduction

Sarcoidosis is a granulomatous interstitial lung disease (ILD) with unknown etiology, characterized by chronic inflammation and mainly affecting the lungs, but with potential systemic involvement [1]. Active T-cell/B-cell cooperation and local production of potentially pathogenic antibodies in the inflamed lung represents a novel pathomechanism in sarcoidosis, and should be considered from both diagnostic and therapeutic perspectives [2]. Actually, the occurrence of autoimmune inflammation as a trigger implicated in sarcoidosis’ development has been reported [3].

Among B-mediated rheumatologic disease, anti-neutrophil cytoplasmic antibody (ANCA)-associated vasculitis (AAV) is a type of histologically granulomatous vasculitis characterized by inflammation affecting small- and medium-sized vessels [4]. AAV can be further subdivided in granulomatosis with polyangiitis (GPA) and microscopic polyangiitis (MPA) [5]. Both diseases involve B cell activation, as well as NK cells, and commonly affect the upper and lower respiratory tract as well as the kidneys [6]. GPA is a necrotizing vasculitis, combining inflammation of the vascular wall with peri- and extravascular granulomatosis (comparable with sarcoidosis).

Immune cells, mainly T cells, are abundant and chronically activated in inflammatory lesions, and a Th1 pattern of cytokines dominates in T cell clones derived from these sites during sarcoidosis and AAV [7,8,9]. In response to this persistent activation, immune cells become exhausted and can no longer produce cytokines or proliferate [10]. This immune exhaustion is partly mediated by the expression of inhibitory co-receptors, known as immune checkpoints (ICs), which belong to the family of negative costimulatory molecules inhibiting T-cell activation, such as programmed death receptor-1 (PD-1) [11]. Since PD-1 can down-regulate T cell responses, higher expression of PD-1 on CD4^+^ and CD8^+^ T cells may down-regulate Th1 cytokines (such as TNF-α and IFN-γ) and decrease mitotic activity so as to induce apoptosis, resulting in resolution of the granuloma [12]. In addition, cytotoxic T-lymphocyte antigen 4 (CTLA-4), mainly expressed on T cells, blocks CD28 costimulation in T cell activation, thus regulating T cell responses [13]. Surface expression of CTLA-4 is strictly regulated in T cells, and failures in the transport mechanism of CTLA-4 to the cell surface can cause susceptibility to autoimmunity [14]. It has been reported that blocking T cell costimulation using CTLA-4Ig might be a useful therapeutic intervention for GPA patients, providing an alternative or complementary approach to conventional treatment with immunosuppressive agents [15].

The T cell immunoreceptor with immunoglobulin and ITIM domain (TIGIT) is another IC receptor expressed on NK and T cell subsets [16]. NK cells with low TIGIT expression had significantly higher IFN-γ expression, degranulation and cytotoxic activity in response to IL-12 stimulation compared to high-level TIGIT expressing NK cells [17,18]. Blocking TIGIT signaling increased both cytokine secretion and cytotoxicity of NK cells [19].

Despite the available evidence in the literature, the role of T and NK cells in GPA and sarcoidosis is still to be determined. The present study aimed to explore the peripheral distribution of IC molecules in order to better elucidate their peripheral tolerance failure, which might reflect the development of diseases.

## 2. Results

### 2.1. Patients

Twenty-three patients were consecutively and prospectively enrolled in the study upon diagnosis, and none of them was on treatment at the time of sampling.

A total of 11 patients (6 females, 4 males, mean ± standard deviation age 57 ± 15.4 years) had AAV diagnoses, and were characterized by active inflammatory process. Six had MPA (three of them had MPA-ILD) and five had GPA; the median duration of disease was 44.1 ± 58.5 months. The median BVAS was 11 ± 6 and the median VDI was 2.5 ± 2.2.

Twelve patients had sarcoidosis (7 females, 5 males, mean ± standard deviation age 56 ± 13.1 years) and they showed Scadding radiological stage II, indicating thoracic lymph node enlargement in addition to sarcoid involvement of the respiratory system.

Ten healthy age- and sex-matched volunteers (6 females, mean ± standard deviation age 55 ± 12.6 years) were enrolled in the study.

### 2.2. Comparison of Immunological Features between GPA, MPA, Sarcoidosis and HC

**Figure 1:**
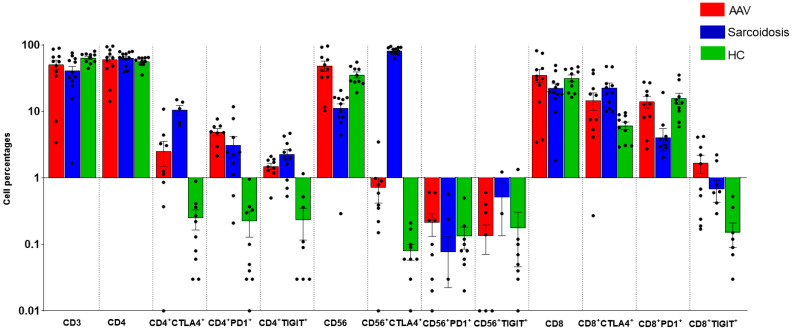
Peripheral distribution of CD3, CD4, CD8 and CD56 cells expressing CTLA4, PD1 and TIGIT in the sarcoidosis, AAV and HC groups. The statistically significant differences between groups are reported in the results section. Abbreviations: AAV, ANCA-associated vasculitis; HC, healthy controls; programmed death-receptor 1, PD-1; cytotoxic T-lymphocyte antigen 4, CTLA-4; T cell immunoreceptor with immunoglobulin and ITIM domain, TIGIT.

Figure 1 shows the peripheral distribution of IC molecules on T and NK cells.

CD3 cell percentages were lower in sarcoidosis than in HC (*p* = 0.0482).

CD4^+^CTLA4^+^ cell percentages were significantly higher in sarcoidosis than in MPA (*p* = 0.0079) and HC (*p* = 0.0117), and numerically higher than GPA (*p* = 0.0482). CD4^+^PD1^+^ and CD4^+^TIGIT^+^ cell subsets were higher in sarcoidosis than in HC (*p* = 0.0250 and *p* = 0.0014, respectively) and higher in MPA than in HC (*p* = 0.0117 and *p* = 0.0113, respectively). CD4^+^PD1^+^ cell percentages were higher in GPA than in HC (*p* = 0.0253).

CD56 cell proportions were lower in sarcoidosis than in HC (*p* = 0.0006) and MPA (*p* = 0.0254). CD56^+^CTLA4^+^ were higher in sarcoidosis than in GPA, MPA and HC (*p* = 0.0085, *p* = 0.0042 and *p* = 0.0004, respectively). CD56^+^PD1^+^ were lower in sarcoidosis than HC (*p* = 0.0462) and GPA (*p* = 0.0360). Small and similar CD56^+^TIGIT^+^ cell percentages were detected in the peripheral blood of patients and controls.

CD8 cell percentages were higher in GPA than in sarcoidosis (*p* = 0.0495). CD8^+^CTLA4^+^ percentages were higher in sarcoidosis than in HC (*p* = 0.0006) and MPA (*p* = 0.0481). On the contrary, CD8^+^PD1^+^ cell percentages were lower in sarcoidosis than in HC (*p* = 0.0042). CD8^+^TIGIT^+^ were higher in MPA than in HC (*p* = 0.0169).

### 2.3. Multivariate Analysis of IC Molecules in Patients and Controls

MANOVA analysis between patients (MPA, GPA and sarcoidosis) and controls associated Lambda (0.004) with a *p*-value (*p* < 0.0001), indicating a risk of erroneously rejecting the null hypothesis < 0.01%.

**Figure 2:**
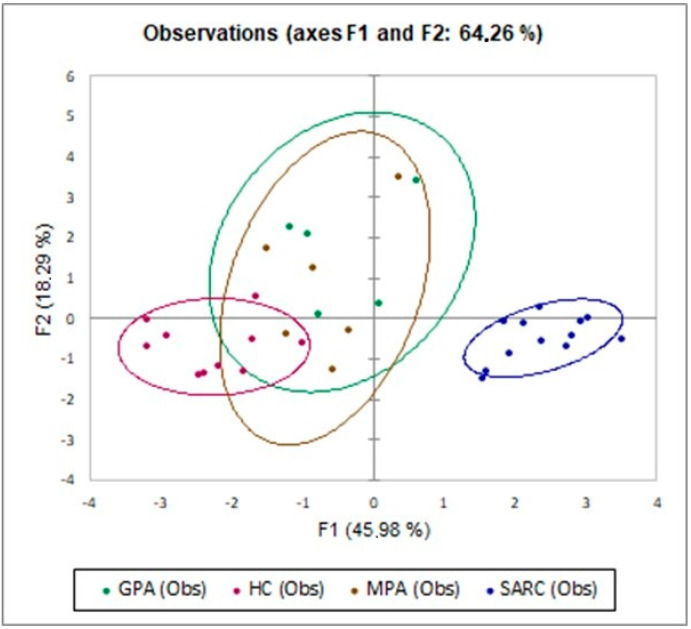
The PCA plot distinguished the disease clusters: MPA, GPA, sarcoidosis and HC showed that they separated on the basis of CD4^+^CTLA4^+^, CD4^+^PD1^+^, CD4^+^TIGIT^+^, CD56, CD56^+^CTLA4^+^, CD56^+^TIGIT^+^, CD8, CD8^+^CTLA4^+^ and CD8^+^PD1^+^. The first and second components explained 45.98% and 18.29% of the total variance (64.26%).

The PCA plot (Figure 2) distinguished the disease clusters: MPA, GPA, sarcoidosis and HC showed that they were separated on the basis of CD4^+^CTLA4^+^, CD4^+^PD1^+^, CD4^+^TIGIT^+^, CD56, CD56^+^CTLA4^+^, CD56^+^TIGIT^+^, CD8, CD8^+^CTLA4^+^ and CD8^+^PD1^+^. The first and second components explained 45.98% and 18.29% of the total variance.

**Figure 3:**
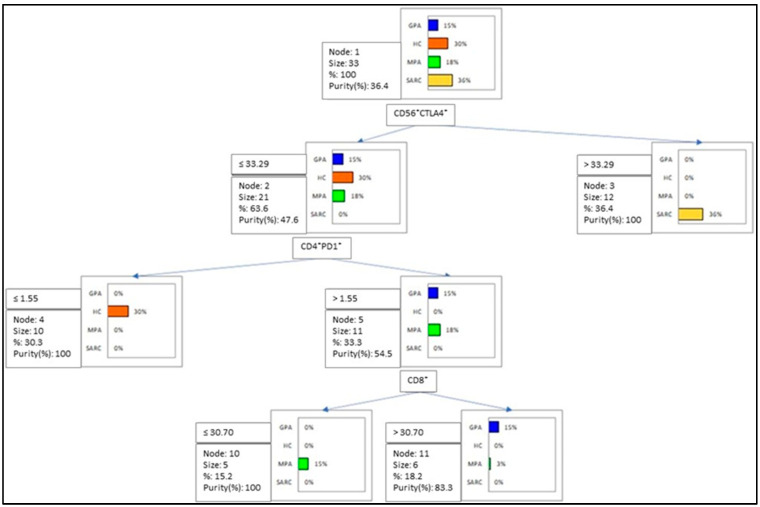
The decision tree model used to cluster the MPA, GPA, sarcoidosis and HC groups according to cut-off values of the cell percentages analyzed in this study. The model showed CD56^+^CTLA4^+^ > 33.29% for 100% of sarcoidosis patients, but CD56^+^CTLA4^+^ ≤ 33.29, CD4^+^PD1^+^ > 1.55% and CD8^+^ > 30.7% for 100% of GPA patients. MPA patients (80%) were classified with CD56^+^CTLA4^+^ ≤ 33.29, CD4^+^PD1^+^ > 1.55% and CD8^+^ ≤ 30.7%, and the model showed CD56^+^CTLA4^+^ ≤ 33.29 and CD4^+^PD1^+^ ≤ 1.55% for 100% of healthy volunteers.

To determine the best clustering variables to distinguish the MPA, GPA, sarcoidosis and HC groups, a decision-tree model was utilized (Figure 3). The model showed CD56^+^CTLA4^+^ > 33.29% for 100% of sarcoidosis patients, but CD56^+^CTLA4^+^ ≤ 33.29, CD4^+^PD1^+^ > 1.55% and CD8^+^ > 30.7% for 100% of GPA patients.

## 3. Materials and Methods

### 3.1. Study Population

In this study, patients referred to Siena Regional Referral Centre for sarcoidosis and Rheumatology Unit of Siena University Hospital were prospectively and consecutively enrolled. Granulomatosis with polyangiitis (GPA) or microscopic polyangiitis (MPA) were diagnosed according to 2022 ACR/EULAR classification criteria [20].

The diagnosis of sarcoidosis was confirmed by multidisciplinary discussion, according to the American Thoracic Society/European Respiratory Society/World Association of Sarcoidosis and other Granulomatous Disorders (ATS/ERS/WASOG) guidelines [21].

All patients with concomitant malignancies and/or under treatment with steroids and/or immunosuppressants were excluded. For each patient, peripheral blood sampling was collected for immunological analysis. Demographic, clinical and radiological features of each patient included in the study were also collected in a pre-structured electronic database. In addition, specific disease score (Birmingham Vasculitis Activity Score (BVAS), Vasculitis Damage Index (VDI)) and sarcoid organ involvement were recorded. Healthy subjects were also enrolled as a control group. They had no history of concomitant pathologies, were not on any medication and had normal lung function test parameters. All patients gave their written informed consent to participate in the study. The study was approved by the regional ethical review board of Siena, Italy (C.E.A.V.S.E. Markerlung 17431 and RHELABUS 22271), and complied with the declaration of Helsinki.

### 3.2. Gating Strategy

**Figure 4:**
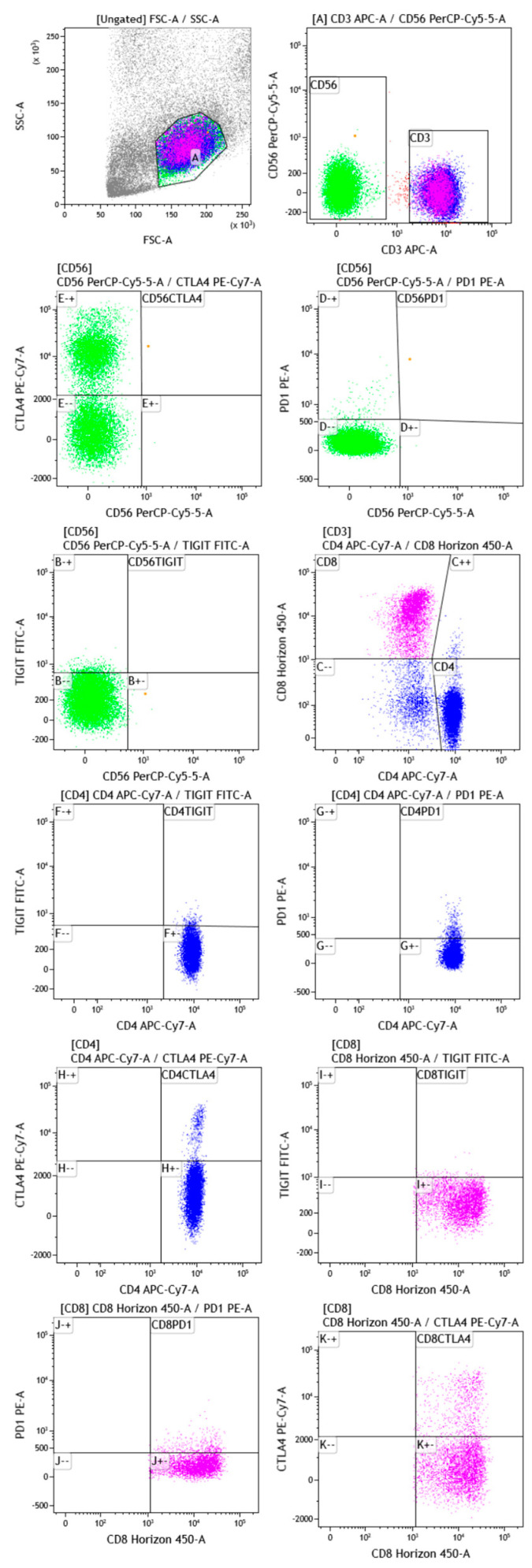
An example of a gating strategy applied for flow cytometry analysis of peripheral blood samples of sarcoidosis and AAV patients, as well as healthy controls. Lymphocytes were distinguished on the basis of forward (FSC) versus side (SSC) scatters, and additional gating was applied to identify CD3^+^ and CD56^+^. Double positive CD56^+^, expressing CTLA, PD1 and TIGIT, was identified. Accordingly, Th and T cytotoxic cells were detected according to CD4 and CD8 markers, respectively. CD4^+^ and CD8^+^, expressing CTLA4, PD1 and TIGIT, were identified. Abbreviations: programmed death-receptor 1, PD-1; cytotoxic T-lymphocyte antigen 4, CTLA-4; T cell immunoreceptor with immunoglobulin and ITIM domain, TIGIT.

Multicolor flow cytometric analysis was performed using the following fluorochrome-labeled monoclonal anti-human antibodies: TIGIT-FITC (MBSA43, Invitrogen, Thermo Fisher Scientific, Waltham, MA, USA), PD1-PE (PD1.3.1.3, Miltenyi Biotec, Bergisch Gladbach, Germany), CD56-PerCPCy5.5 (5.1H11, BioLegend, San Diego, CA, USA), CTLA4-PeCy7 (14D3, Invitrogen, Thermo Fisher Scientific), CD3-APC (OKT3, BioLegend), CD4-APCCy7 (REA623, Miltenyi Biotec) and CD8-BV421 (REA734, Miltenyi Biotec). Cells were stained for 30 min at 4°C, measured with a Facs CantoII flow cytometer and analyzed using Kaluza Analysis 2.1 (Beckman and Coulter Life Sciences, Brea, CA, USA). The gating strategy is reported in Figure 4.

### 3.3. Statistical Analysis

All values were expressed as median and Interquartile Range (IQR) and mean ± standard deviation, when appropriate. The normal distribution of values was determined with the Shapiro–Wilk test. Non-parametric one-way ANOVA (Kruskal–Wallis test) and the Dunn test were performed for multiple comparisons. For categorical variables, Fisher’s exact or Chi-squared tests were used to compare proportions between groups. Spearman’s test was used to find correlations between clinical and immunological parameters.

Multivariate analysis, adjusted for sex and age, was performed on cell subsets (CD4^+^CTLA4^+^, CD4^+^PD1^+^, CD4^+^TIGIT^+^, CD56, CD56^+^CTLA4^+^, CD56^+^PD1^+^, CD8, CD8^+^CTLA4^+^, CD8^+^PD1^+^ and CD8^+^TIGIT^+^) that differed in a statistically significant manner between the sarcoidosis, MPA, GPA and healthy control (HC) groups. MANOVA analysis was performed to determine any significant effects of sarcoidosis and AAV diagnosis with respect to the control group (explanatory variables: GPA, MPA and sarcoidosis versus HC) considered in interaction or otherwise with CD4^+^CTLA4^+^, CD4^+^PD1^+^, CD4^+^TIGIT^+^, CD56, CD56^+^CTLA4^+^, CD56^+^PD1^+^, CD8, CD8^+^CTLA4^+^, CD8^+^PD1^+^ and CD8^+^TIGIT^+^ (dependent variables). The null hypothesis, which excludes any effect of the explanatory variable on the combination of dependent variables, i.e., that MPA/GPA/sarcoidosis diagnosis has/have no effect on the dependent variables, was assessed, with a very small risk of being wrong.

Supervised Principal Component Analysis (PCA) was used in an explorative approach to identify trends in immunological features by 2D representation of the multi-dimensional data set.

To investigate the best thresholds for classifying MPA, GPA, sarcoidosis and HC, four groups were formed. A classification and regression decision tree were constructed to determine the best clustering variables according to the Gini criterion. We created a series of test/training partitions to evaluate the accuracy of potential binary classifiers by means of a confusion matrix.

A *p*-value less than 0.05 was considered statistically significant. Statistical analysis was performed by GraphPad Prism 9.4 and XLSTAT 2021 software.

## 4. Discussion

The present study analyzed, for the first time, the CTLA4, PD1 and TIGIT expression on T and NK cell subsets in the peripheral blood of sarcoidosis, MPA and GPA patients.

Increased expression of CTLA4 and TIGIT, along with decreased expression of PD1, was reported on the T and NK cells of sarcoidosis patients compared with AAV patients and the HC group. According to cell subsets analyzed through flow cytometry in our patients, the PCA analysis defined three clusters (sarcoidosis, HC and AAV) of diseases, with a total variance of 64.26%. Peripheral lymphopenia was described in sarcoidosis patients, according to the literature data. CTLA4^+^NK cells clustered for 100% of sarcoidosis patients, according to the decision tree analysis, while PD1^+^CD4 and CD8 cells clustered for 100% of GPA patients.

The pathogenesis of AAV can be influenced by several factors, including genetics (associated with major histocompatibility antigens and other immunoreactive molecules), infections and environmental exposure to physical and chemical agents. The development of disease is also influenced by hormones and stressful life events. AAV is characterized by the presence of an inflammatory milieu, leukocytes and acute-phase proteins. This suggests that comparative studies should be conducted to better understand the peripheral tolerance failure associated with sarcoidosis and AAV. Despite emerging evidence that AAV and sarcoidosis are related with regard to dysregulation of the cellular immune system [22,23], there are currently no data regarding the adaptive immune cell profiling common to both diseases, also considering IC molecule expression on CD4, CD8 and NK cells.

The role of the immune system in such diseases’ pathogenesis include the loss of tolerance of T and B cells [24]. Inhibitory pathways, denoted as IC molecules, normally restrain T-cell function and maintain peripheral tolerance, keeping auto-reactive T cells anergic [25,26]. Along with this, granulomatous inflammation is found in affected organs of sarcoidosis and GPA patients, and is characterized by T-cell dependent granuloma formation [27].

T cell dysfunction occurs when there is chronic exposure to antigens, such as in the context of autoimmunity and cancer [28,29]. This loss of function is referred to as T cell exhaustion profile, and is characterized by increasing expression of inhibitory receptors such as PD1, CTLA4 and TIGIT [30,31]. Our study described, for the first time, increased PD1, TIGIT and CTLA4 expression on CD4 cells of sarcoidosis patients. These are key components of sarcoid granuloma and are chronically activated; therefore, our findings are intriguing, since they suggest that a dysregulation of IC molecules may significantly contribute to the chronicization of granulomatous sarcoid inflammation, which is a critical issue in terms of response to treatment and prognosis.

T cell exhaustion includes the loss of IL-2 production, decreased proliferative capacity and reduced cytotoxic abilities, including impairment in granzyme B, IFN-γ and TNF-α production [10,31]. The increased expression of PD1 may down-regulate Th1 cytokines (such as TNF-α and IFN-γ) and decrease mitotic activity so that it induces apoptosis, which leads to the resolution of the granuloma. As noted by Wilde et al., we detected high levels of PD1 expression on peripheral CD4 cells in our GPA patients in comparison to the HC group, which counterbalanced the persistent T-cell activation [32].

Along with this, TIGIT belongs to the second wave of immune-checkpoint receptors and works in synergy with PD-1 [30,33]. It is reported to be highly expressed in tumor-infiltrating T lymphocytes in the lungs. TIGIT expression was higher in CD4-positive cells of sarcoidosis patients than in the controls, confirming our previous results [12]. This result was further confirmed in the present study.

Notably, the inhibitory receptors CTLA-4 and PD-1 act to constrain autoimmunity, and their inhibition has been reported to initiate or exacerbate sarcoid-like inflammation, reinforcing the notion of altered immune activation and, possibly, an autoimmune component in sarcoidosis [12].

Recently, our group analyzed the CTLA4 expression on T and NK cell subsets in three different anatomical compartments (bronchoalveolar lavage, lymph nodes and peripheral blood) of sarcoidosis patients, reporting peripheral CD4^+^CTLA4^+^ as the best cluster variable to distinguish sarcoidosis patients from healthy volunteers [12]. Accordingly, in the present study, CTLA4 on peripheral NK cells was identified as the best clustering variable for sarcoidosis patients, rather than for HC, MPA and GPA patients.

The importance of NK cells in this setting is indirectly confirmed by the fact that ipilimumab (anti-CTLA-4 monoclonal antibody) treatment depletes regulatory T cells in cancer patients, and is reported to improve NK cells’ functions in advanced cutaneous melanoma patients [34]. According to the literature data, NK cells were significantly depleted in sarcoidosis, with respect to other ILDs that might play a protective role in the pathogenesis of such disease [35]. Activation of the CTLA-4 receptor can inhibit IL-2 production in CD4^+^ T cells, and a blockade of CTLA-4 improved NK cell cytotoxicity in an indirect way by ensuring the restoration of NK cell functions [36]. Thus, it could be a potential therapeutic strategy for sarcoidosis patients.

## 5. Conclusions

The PD1, CTLA4 and TIGIT expression on T and NK cells negatively regulates the immune function of T-cells. Previously neglected cells, including NK cells, have been studied more recently for their contribution to sarcoid granuloma formation. Our study provides innovative and intriguing insights into the pathogenesis of sarcoidosis, showing a reliable dysregulation of expression of IC molecule in different immune cells which may significantly contribute to the formation and chronicization of granuloma. Moreover, our analyses showed substantial differences between sarcoidosis and AAV, further confirming the immunological peculiarity of this disease. Despite these advances, the pathogenesis remains incompletely understood, indicating an urgent need for further research to reveal the distinct immunological events in this process. There is hope to open up new therapeutic avenues, and, if possible, to develop preventive measures.

## Data Availability

The data presented in this study are available upon request from the corresponding author.

## Abbreviations

AAV: ANCA-associated vasculitis; ILD, interstitial lung disease; PD1, programmed death receptor-1; CTLA-4, cytotoxic T-lymphocyte antigen 4; TIGIT, T cell immunoreceptor with immunoglobulin and ITIM domain; CD-, cluster of differentiation; Th-, T helper; GPA, granulomatous with polyangiitis; MPA, microscopic polyangiitis.
